# Screening and Invasive Testing in Twins

**DOI:** 10.3390/jcm3030865

**Published:** 2014-07-29

**Authors:** Giovanni Monni, Ambra Iuculano, Maria Angelica Zoppi

**Affiliations:** Department of Obstetrics and Gynecology, Prenatal and Preimplantation Genetic Diagnosis, Fetal Therapy, Ospedale Microcitemico, via Jenner, 09121 Cagliari, Italy; E-Mails: ambraiuc@tiscali.it (A.I.); angelica.zoppi@tiscali.it (M.A.Z.)

**Keywords:** twins, screening, invasive diagnosis, therapy

## Abstract

Prenatal screening and testing for trisomy 21 in twin pregnancies poses a number of challenges: the exact estimate of the *a priori* risk of trisomy 21, the choice of prenatal screening test and/or invasive techniques to employ for the diagnosis and the impact of the result on the options of treatment in case of discordant results within a twin pair or among multiples. These different aspects are discussed below while recognizing that many issues remain unresolved.

## 1. Introduction

In twin pregnancies, the entire course of prenatal genetic screening and diagnosis poses a number of unusual concerns, and screening is rather more complicated than in singleton pregnancies [1,2].

Prenatal testing for trisomy 21 in twins implies a higher frequency of adverse neonatal outcomes, particularly due to the increased rate of preterm births. The future parents involved in such pregnancies, however, are generally predisposed to increased psychological optimism [3,4].

Despite the increased frequency of twin pregnancies and the intensified interest in the prenatal diagnosis of multiple gestations in recent years, many considerable challenges remain.

The first challenge is to establish the exact estimate of the *a priori* risk of trisomy 21, followed by the determination of which prenatal screening test to use and which invasive techniques to employ for the diagnosis, as well as the impact of the possible result. There are a number of possible treatment options available in the case of discordant results within a pair of twins or among multiples.

## 2. Estimate of the *a Priori* Risk

### 2.1. Making the Diagnosis of Twin Pregnancy

It is crucial to recognise the twin or multiple pregnancy as early as possible to prepare for the subsequent course of tests and diagnoses. The early diagnosis of multiple gestations is far from accurate, and it has been reported that at least 40% of twin pregnancies are not recognised until 13 weeks of gestation [5].

The major errors in screening and diagnosis can include the underestimation of an ongoing twin pregnancy (“the appearing twin”) or the misdiagnosis of an ongoing singleton pregnancy as one that started as a twin pregnancy or more (“the vanishing twin” phenomenon) [6,7].

Gestational sacs can be visualised by transvaginal ultrasound at 35 days after the last menstruation in 95% of cases, whereas foetal cardiac activity can be visualised at 44 days [8]. The visualisation of more than one yolk sac inside the gestational sac enables us to accurately define the pregnancy as multiple. Amniotic membranes are detectable by 7.0 weeks [9,10]. The close monitoring of biochemical markers of pregnancies achieved by ART often leads to the early suspicion of a multiple pregnancy even when multiple gestational sacs are not yet detectable, as the mean beta-hCG value in multiple gestations is significantly higher than that in singleton pregnancies [11].

Underestimating the number of foetuses occurs frequently, particularly if relying on an early assessment. A scan performed as early as 5.0–5.9 weeks may undercount the number of embryos; in one study, 24 (11%) of 213 dichorionic twin gestations were initially undercounted as singletons, as were six (86%) of seven monochorionic twin gestations, with respect to scans performed at 6.0 weeks or beyond in which an “appearing twin” was detected [6]. In a recent series of 48 monochorionic twin pairs for whom the earliest scan was not performed until 11.0 weeks, undercounting occurred in 18 of 48 (37.5%) cases [12]. Monochorionic twin pregnancies occur relatively less frequently and are less expected; together with the appearance of the amnion inside the gestational sac after 7.0 weeks, this may explain the tendency towards a relatively higher underestimate of monochorionic pregnancies. However, the finding of an “appearing foetus” in a monochorionic pregnancy may correlate to a “higher risk” for the pregnancy and predict cases at higher risk for major pregnancy complications within this class of pregnancies [12].

The arrest of development and the subsequent reabsorption of an embryo may occur in early gestational life and is termed “vanishing embryo” syndrome [13]. Regarding the frequency of this phenomenon, very different figures are employed to define the pregnancy, with estimations based per patient, per gestational sac, per embryo or cardiac activity [14]. If two sacs are identified sonographically in the first trimester, the loss of one twin can be expected in 27.1% of pregnancies achieved by assisted reproduction techniques (ART) and in 40.5% of spontaneous pregnancies [15]. If two embryos have been detected, spontaneous embryo reduction has been reported to occur in 38% of cases when the pregnancy was achieved by ART and in 7.3% of spontaneous pregnancies [15].

In subsequent surveys, further aspects of the modality of achieving pregnancy have been explored in relation to the outcome of pregnancies [16]. While the risk of fetal loss, extended preterm delivery and neonatal/infant death is, generally, significantly higher among monochorionic twins rather than dichorionic, the spontaneously conceived twins, either monochorionic or dichorionic, are more likely to suffer a complicated outcome than twins resulting from the treatment of infertility [17].

The “vanishing twin” condition may have some implications for screening and invasive prenatal diagnosis, and it is important to consider it, rather than leave it unrecognised.

If ultrasound screening shows that there is a second sac containing a non-viable foetus, an alteration of the maternal biochemical markers many weeks later is possible [18,19], and this fact must be considered in the screening of such a pregnancy continuing as a singleton.

Furthermore, the vanishing of an embryo has been suggested to explain the discordance between chorionic villus karyotype and foetal phenotype [20]. The vanishing twin phenomenon has been used to explain discrepant chromosome results [21]. It has also been suggested that the vanishing twin phenomenon may be responsible for some cases of isoimmunisation during pregnancy in which a rhesus-positive foetus disappears in a previously unsensitised rhesus-negative mother [15].

The “vanishing twin” phenomenon must also be considered when counselling patients to make a decision about multifoetal pregnancy reduction (MFPR), regarding the timing of the procedure in particular. Spontaneous reabsorption, which occurs earlier in gestation, may eliminate the need for reduction in some cases. Given the low probability of spontaneous embryonic demise later in the first trimester, however, the majority of procedures are performed at 10–13 weeks [22,23].

### 2.2. Making a Non-Invasive Prenatal Diagnosis of Monozygosity or Dizygosity

Procedures involving the direct collection of foetal cells are invasive, but they offer a definitive prenatal assessment of zygosity. The routine non-invasive method used to prenatally discriminate between monozygotic and dizygotic twins is ultrasound based. This method is non-invasive but also non-definitive. Attention should be placed on the determination of chorionicity by first-trimester ultrasound examination of the foetal placenta and membranes, by the identification of the “λ” sign (in dichorionic twins) and the “T” sign (in monochorionic twins) [24,25]. Approximately 80% of twin pregnancies are dichorionic, of which approximately 10% are monozygotic, and the remaining 20% are monochorionic. Monochorionicity may be considered a clear sign of monozygosity. Regarding dichorionic twins, all the cases in which the foetuses are of different sexes can be assumed to be dizygotic [26]. The diagnosis of foetal sex by ultrasound, while non-definitive, can be obtained with good predictive success in the first trimester (at 14.0 weeks the success rate is about 90%) [27].

In dichorionic pregnancies that are the result of IVF-ET (*in vitro* fertilisation and embryo-transfer), with foetuses showing the same sex, in the case of multiple embryo transfer (MET), the percentage of dizygotic twins is very high, at 99.3% (monozygotic: 0.7%) [28]. By contrast, in the case of a single embryo transfer (SET), the percentage of monozygotic twins must be 100% [28]. In spontaneous dichorionic pregnancies with foetuses of the same sex, the percentage of dizygotic twins should be calculated considering some maternal factors, such as age and ethnicity, as dizygotic pregnancies are more frequent among older patients and Afro-Caribbeans.

Recent studies using maternal plasma DNA sequencing have shown the possibility of the non-invasive prenatal determination of twin zygosity [29,30].

### 2.3. How to Calculate the Risk of Trisomy 21

In singleton pregnancies, the foetal risk rate for trisomy 21 is usually based on the maternal age at term and can be expressed at various times in pregnancy by correcting for the higher rate of spontaneous loss of affected pregnancies.

In twin monozygotic pregnancies, the risk of both foetuses being affected is similar to the maternal-age risk (*i.e.*, 1 in X, where X is the risk related to maternal age), while the risk of only one foetus being affected is virtually null. Therefore, in monozygotic pregnancies, the risk is per pregnancy.

In dizygotic pregnancies, the risk could be expressed per foetus and/or per pregnancy.

For dizygotic pregnancies, special algorithms for calculation have been formulated [31,32].

A considerable challenge is to quantify in real life the frequency of foetuses and new-borns with trisomy 21 in twin pregnancies. For a long time, the custom has been to refer to the frequency of trisomy 21 in singletons or to rely on complex probabilistic calculations.

In dizygotic pregnancies, traditional algorithms consider the foetal risk as the same as that for maternal age (*i.e.*, 1 in X), while the risk of both foetuses being affected as very low (*i.e.*, 1 in X multiplied by 1 in X, or 1 in X^2^). At the same time, in dizygotic pregnancies, the risk of only one foetus being affected is considered approximately 2 times 1 in X (this is a high “pregnancy risk”), and there is another possibility to consider, *i.e.*, the risk of at least one foetus being affected (that is, the sum of the probability of either having both foetuses affected or having one affected), which is approximately 2 times 1 in X [32]. As a result of these calculations, the idea that the risk of trisomy 21 in twin pregnancies is higher than that of a singleton pregnancy persisted for a long time (the risk at age 31 was considered to be equal to that of a 35-year-old woman) [33]. More recently, considering the difficulties in very accurately estimating the birth prevalence of trisomy 21 in twins because of its assessment mode (during pregnancy, at birth or later) and the rate of intrauterine demise of affected foetuses among twins, the actual frequency of trisomy 21 observed in new-borns has been considered to be similar to the frequency in singletons or even lower [34,35]. A very recent population-based study of the prevalence of the Down syndrome in multiple pregnancies from European registries has shown the risk per foetus/baby to be lower in multiple than in singleton pregnancies (the relative risk of Down syndrome babies, adjusted for maternal age, is 0.58 times in multiple births compared to singleton ones) [36]. The analysis was next performed dividing the pregnancies in monozygotic and dizygotic. The relative risk for monozygotic pregnancy was 0.34 times in comparison to singleton, while for dizygotic pregnancies the relative risk of at least one co-twin being affected by Down syndrome was 1.34 times compared to the risk of singleton pregnancies [36].

### 2.4. Dating the Pregnancy Discordance of Crown-Rump Length between Twins

The risk for trisomy based on the gestational age can be calculated according to the last menstrual period or, if the dates do not correspond (and such a result is almost always followed with screenings using biochemical markers), according to the gestational age derived from the Crown-Rump Length (CRL). In a twin couple either monochorionic or dichorionic, there is evidence that the CRL of the two foetuses frequently does not exactly match, with the mean difference among CRLs being approximately 3.4 mm, and the mean percentage difference of the bigger foetus being 5.1% of CRL. In 95% of cases, the CRLs differ by less than 10 mm in length, or up to 15% of the greater length of the foetus and 3–4 days of gestation [37]. This makes it difficult to date either mono- or dizygotic pregnancies according to the CRL, as it would lead to having two foetuses with different gestational ages within the same pregnancy. As the phenomenon of superfoetation in humans is assumed to be very rare, although possible [38], it is agreed that in twin pregnancies, the gestational age is the same for both foetuses. Therefore, the reference values obtained from singleton pregnancies are used for dating twin pregnancies. Another issue to consider has been previously discussed [39]: If we must date the pregnancy based on the CRL, in the case of discordant CRLs, which foetus should we choose for the dating—the one with the greater CRL or the one with the smaller CRL? The earlier studies suggest to date using the foetus with the greater CRL [40,41]. Furthermore, we must consider that a shorter CRL in a twin pregnancy can be a sign of a chromosomal anomaly or malformation in that foetus, and in any case, it is a negative prognostic sign [42,43].

However, another suggestion is to base the dating on the smaller twin after it has been revealed that dating is more accurate if based on the smaller twin in ART pregnancies. The larger foetus is to be used for dating only if the difference between the CRLs exceeds the 95th percentile [37].

## 3. Which Prenatal Screening Test Should be Used?

### 3.1. Nuchal Translucency in Twins. Different Management of Monochorionic and Dichorionic Pregnancies

Many issues regarding the estimate of the *a priori* risk of trisomy 21 in a twin or multiple pregnancy remain unresolved. Ultrasound and biochemical markers in pregnancy make the *a priori* risk even harder to define.

Nuchal translucency (NT) measurement can be used for screening in twin and multiple pregnancies because twin pregnancy foetuses with trisomy 21 may show a thickening of the NT. However, there are many peculiarities that make the measurement of twin pregnancy NT rather complicated and generally less accurate, with “accuracy” implying here the combination of the sensitivity and specificity of the test. Monochorionic twins tend to have a higher percentage of increased translucency compared to dichorionic twins, and the nuchal translucency thickness is, on average, significantly higher [44,45]. This fact appears to be related to placental structure rather than zygosity. Many placental connections are actually present in all pairs of monochorionic twins and can therefore have an effect on first-trimester NT thickness. However, because there is no evidence of a higher incidence of trisomy 21 in monochorionic twins, it is important to consider this phenomenon to avoid unnecessary invasive procedures for verification [46]. Enlarged NT in monochorionic twins may instead indicate heart defects, malformations, twin-to-twin transfusion or an early placental fluid imbalance without subsequent complications [43,47,48,49,50].

Regarding the inter-twin NT difference in a twin couple, the bigger difference is observed in monochorionic twins, followed by dichorionic dizygotic twins and, last, by dichorionic monozygotic twins [46]. This distribution shows that the imbalance of placental fluids present in identical twins and the normal individual differences that are expected in the pairs of fraternal twins may underlie this phenomenon.

As far as the integration of the NT value into the calculation of the trisomy 21 risk for monochorionic twins is concerned, after the initial period in which the use of the thicker NT was preferred [51], it became evident that the most effective screening method for trisomy 21 is using the average NT measures of the two foetuses [41]. The average NT in this case is the arithmetic mean. Another suggestion, which seems to better interpret the result, is to use the geometric mean [52], although others use the average of the risk calculation in the two foetuses [53]. This issue still requires a larger consensus.

Dichorionic twins are assumed to be dizygotic, and the individual NT is integrated in the background risk. The most frequent practice is to calculate the risk per foetus and not per entire pregnancy. However, a correlation between the NT of a pair of twins has been found, both in monochorionic and dichorionic couples [28,46,54,55]. This information has some implications regarding the risk calculation in dizygotic twin pregnancies, where the NT value of the co-twin should not be ignored. In fact, when both NT values are increased in a pair of dizygotic twins, it is more likely that such an increase is rather the expression of a familial tendency towards enlarged NT thickness rather than the rare occurrence of a chromosomal abnormality of both foetuses, and this reduces its significance in modifying the background risk for trisomy 21 in that pair of twins [56].

### 3.2. Other Ultrasound Markers in Twins

No differences regarding the possibility of assessment in monochorionic *versus* dichorionic couples have been reported regarding the nasal bone and the possibility of using this parameter in multiples [57]. However, such assessment is relatively more difficult, and studies have described lower sensitivity for aneuploidies among twins rather than in singletons [58].

Tricuspid valve regurgitation and ductus venosus are two markers for trisomy 21 that involve evaluation of the foetal blood circulation. For the ductus venosus in dichorionic foetuses, the results are similar to those of singleton foetuses [59].

In monochorionic foetuses, however, a possible imbalance in placental or foetal circulation may affect these two markers, and the identification of foetuses with trisomy 21 may be less accurate.

A condition of temporary cardiac or placental imbalance, or early signs of twin-to-twin transfusion, may present with a reverse a-wave in ductus venosus [48,59], whereas tricuspid regurgitation is more frequent in foetuses with congenital heart defects, for which monochorionic pregnancies are typically at higher risk.

### 3.3. Biochemical Markers in Twins. Different Management for Monochorionic and Dichorionic Pregnancies

In singletons, free-beta hCG and PAPP-A are usually incorporated into a test that considers the risk relative to maternal age, gestational age and NT thickness. In the management of the combined test, it should be first established if the twin couple is dichorionic or monochorionic. Initially, not enough data was available regarding certain biochemical serum markers in twin pregnancies with trisomy 21 and calculating the adjusted risk was difficult, therefore it was called “pseudo-risk” [60]. The concentrations of the biochemical markers in twin pregnancy are approximately twice those in singletons because the biochemical data may be, in part, the expression of the placental volume. However, in normal foetuses, there is a difference between the marker concentrations in the two types of twins, as the free-beta and PAPP-A concentrations in dichorionic twins are approximately 2 MoMs of individual normal singletons and approximately 1.5 MoMs of those in monochorionic twins [60,61,62,63,64]. Furthermore, studies performed *in vivo* that have assessed placental volume by 3D ultrasound did not observe a substantial difference (measured by the VOCAL technique) between monochorionic and dichorionic pregnancies, although a significant difference between single and twin pregnancies was evident [65]. The scarcity of reference values for free beta-hCG and PAPP-A for twin pregnancies affected by trisomy 21, either monochorionic or dichorionic/dizygotic, is an important issue, as the majority of studies have been performed using reference values obtained from singleton pregnancies.

In the situation where both twins are affected (e.g., most monozygotic cases), it would be expected that the alteration in serum analyte concentrations typically seen in affected singleton pregnancies would be proportionately altered in the twin affected pregnancies. However, when only one of the two foetuses is affected, the alteration in the serum marker concentrations would be less apparent.

The efficacy of serum screening in dizygotic twin pregnancies (where usually at most only one foetus would be affected) would therefore be expected to be lower than that seen for singletons.

A further difficulty has been that there has been very little data available for the serum markers in pregnancies where one or both foetuses are affected. It was expected that these affected pregnancy serum marker concentrations are higher (because there are two foetal/placental sources contributing), that they would be proportionate to that seen in singleton pregnancies, and that the affected medians could therefore be estimated from the values seen in singleton pregnancies. For the purposes of calculating risk using these estimated medians, two approaches are in use. The first adjusts the concentrations seen in a twin pregnancy to those seen in a singleton pregnancy and computes a “pseudo-risk” based on those values. The second approach directly computes the risk from the observed concentrations relating them to those expected for twin pregnancies. The use of a pseudo-risk should limit the number of false-positive results but does not provide a true calculation of risk [66].

Whichever method is used, it should be recognized that the screening is generally less effective in twin pregnancies, it is based on scant data and assumptions for the generation of the statistical parameters for affected pregnancies, and there are also uncertainties about the prior risk used in the calculations.

Such an uncertain basis generates imprecision, and this is why extensive application in the routine practice of the combined test in twin pregnancies, outside a study context, requires considerable caution.

In addition, it should be considered that a significant number of twin and multiple pregnancies result from ART, which implies other major concerns regarding the use of biochemical markers, although not excluding their use completely.

For this reason, there are some practice guidelines for the screening test for trisomy 21 in the first trimester, while not excluding the combined test, it is generally emphasised that its efficiency is low and that it is not as accurate as desired to enable patients to make appropriately informed decisions about the pregnancy [67].

The combined test (because of the biochemistry) is not recommended for pregnancies with more than two foetuses.

### 3.4. Application of NIPT to Multiple Pregnancies

Non-Invasive Prenatal Testing (NIPT) by analysis of cell-free DNA (cfDNA) in maternal blood has shown a high detection rate for trisomy 21 among other frequent autosomal trisomies in an unselected population of first trimester singleton pregnancies [64]. The possibility of applying the NIPT to twin pregnancies is a topic addressed only very recently. The initial results indicate the ability of this method to distinguish cases with affected foetuses in twin pregnancies [68,69].

A distinction between monozygotic and dizygotic pregnancies should be made as well. In monozygotic pregnancies, where the twins have identical genomes, the NIPT is practiced as in pregnancies with singleton foetuses and appears to be better than or at least as efficient as in singletons.

For pregnancies with dizygotic twins, different problems may arise instead. The two foetuses, which are not identical and may be discordant for aneuploidy, may release different amounts of foetal DNA into the maternal blood, and the test might therefore be inaccurate if the amount produced by the affected foetus is too low. In a dizygotic pregnancy discordant for trisomy, if the foetal fraction of the affected foetus is below 4%, which is the percentage requested in a singleton pregnancy to detect aneuploidy, the results may incorrectly indicate a low risk. Therefore, to obtain a reliable diagnosis in a dizygotic pregnancy, it has been proposed that the lower fraction of the two foetuses should be estimated in the assessment of the risk of aneuploidy rather than the total foetal fraction. NIPT in the first trimester screening can be offered either to all patients at 10 weeks and then followed by the nuchal translucency scan at 12 weeks or as a part of a contingent strategy after receiving the result of the first-line screening [70,71].

## 4. Invasive Diagnosis

Invasive procedures for prenatal diagnosis in twin pregnancies must contend with certain peculiarities that are specific to this type of pregnancy.

In this field, we must first distinguish between monochorionic and dichorionic twins, and this must be performed first because it clearly defines the choice between the two possible pathways. Regarding chorionic villus sampling (CVS), the approach could be transabdominal (TA), transcervical (TC) or both combined, and sampling error/cross-contamination are important issues. Regarding amniocentesis, single uterine entry *vs.* double uterine entry, sampling error/cross-contamination and the use of dye are matters to be considered [72,73]. The rate of pregnancy loss due to the invasive nature of the procedure appears to be higher in twin pregnancies than in singleton ones. However, it is important to note that the background risk of miscarriage is also higher in twins, and therefore, the results of singleton and twin pregnancies are not directly comparable [74].

A meta-analysis of nonrandomised cohort studies has shown that the total pregnancy loss may be interpreted as similar for CVS and amniocentesis in twin pregnancies (3.84% for CVS and 3.07% for amniocentesis). It is important to note that none of the CVS studies has considered the chorionicity when reporting the miscarriage rate after any invasive test. However, there are a few studies regarding procedure-related pregnancy loss following amniocentesis that have considered the chorionicity [75,76,77].

There is a common concern regarding an increase in the risk of adverse outcome with the number of uterine entries, although it is not statistically significant [78,79].

The single uterine-entry technique requires visualisation of the dividing membrane to advance the needle tip into both sacs under direct ultrasound vision; otherwise, a second puncture should be performed.

Regarding other methods of foetal sampling, cordocentesis and intrahepatic umbilical vein sampling should be considered [73]. These techniques allow specifically sampling each foetus and are used as second-level procedures in selected cases (*i.e.*, mosaicisms or the failure of previous procedures) [79]. Cordocentesis carries a sampling success rate that is similar in singleton and twin gestations (98.8% *vs.* 97.3%), the foetal loss rate within 2 weeks of cordocentesis is also similar (1.4% and 1.1%, *p* = 0.42) [79].

### 4.1. Monochorionic Pregnancy

As monochorionicity and monoamnionicity indicate monozygosity, the expected condition is that the two foetuses will exhibit an identical karyotype [80]. With this assumption, a single chorionic villus sampling in the first trimester or a single sampling of amniotic fluid at a later gestational age can be rationally justified. This has a particular importance because it is evident that the introduction of the needle through the maternal abdomen to obtain foetal material for prenatal diagnosis twice leads to greater risks than does a single introduction.

Cases of discordant karyotype (heterokaryotypia) are reported with increasing frequency in monozygotic twins. These events may be due to errors occurring during mitosis or twinning events, such as the phenomenon of postzygotic non-disjunction. This condition should be considered in cases in which discordant sonographic signs are found, such as an increased nuchal translucency or a malformation [81,82]. In these cases, it is essential to sample both foetuses and that each sampling is attributed with certainty to each foetus. Because chorionic villus sampling cannot guarantee such certainty, it is generally preferred to sample amniotic fluid from each twin sac. Genotyping with microsatellite markers is used to confirm the monozygosity of discordant twins, and cytogenetic studies may benefit from molecular karyotyping in cases of discordant phenotypes between monozygotic twins [83].

However, it must be considered that for the diagnosis of genetic diseases with DNA analysis, it is preferable to sample the chorionic villi and then proceed with this technique in monochorionic pregnancies when the risk is very high [73].

Obtaining amniotic fluid from both sacs in a monochorionic twin pregnancy is not always a simple procedure. In fact, in approximately 20% of cases, there may be a significant reduction in the amniotic fluid in one of the two sacs due to the occurrence of TTTS, which may result in a huge discordance of fluid between sacs or in a moderate discordance of fluid, associated or not with a selective intrauterine growth restriction, which may complicate the sampling of both amniotic sacs. Injection of indigo carmine into the first sampled amniotic sac may avoid sampling errors, which are frequent in cases of folding (approximately 30% of cases) or overlapping of the intertwin membrane, where iatrogenic septostomy may indeed occur [84,85].

Amniotic fluid sampling for invasive diagnosis can sometimes be obtained with the amnioreduction during an invasive procedure for the treatment of TTTS before laser treatment.

### 4.2. Dichorionic Pregnancy

For dichorionic twins, monozygosity is expected in approximately 10% of cases, with most pregnancies being dizygotic. Therefore, the choice of diagnostic procedure is governed essentially by the type of placentation.

It is possible to properly sample each foetus by chorionic villus sampling in the first trimester.

The risk of cross-contamination with CVS, which is unique to a twin pregnancy, is approximately 1%. In the case of amniocentesis, an intra-amniotic dye can be used [73].

Chorionic villus sampling is usually performed between the 10th and 12th week of gestation. To achieve the sampling of both placentas, different modes of sampling can be used, including transabdominal, transcervical and transvaginal, with the latter two being primarily used in combination. The transcervical and transvaginal modes can be performed until the 13th week of gestation, whereas the transabdominal method has the advantage that it can be performed later as well [73]. The sampled placental material can be used for cytogenetic studies, molecular karyotyping and genotyping to exclude monozygosity or erroneously repeated sampling of the same foetus. If the result is discordant, with evidence of a karyotype abnormality in one foetus, the type of placentation allows the therapeutic option of first-trimester selective embryo reduction.

Amniocentesis, with the use of indigo carmine in the first sampled sac, can be performed from the 15th week on, and this allows a mid-trimester selective reduction in the case of discordant results with the affected foetus.

## 5. Selective Termination

The diagnosis of discordant karyotype, with the presence of a serious anomaly in a pair of twins may be the reason for the request by a pregnant woman for selective termination of the affected foetus. A further reason is the presence of a structurally anomalous foetus, which may increase the risk of adverse perinatal outcome of the whole pregnancy [86].

Selective termination is considered a relatively safe technique, but there are some important aspects to consider: the procedure-related risk of pregnancy loss, the possibility of maternal coagulopathy due to the prolonged retention of a nonviable foetus, and psychological effects on the mother who is carrying a non-viable foetus for a long period during the gestation.

Multifoetal pregnancies (more than two) and high-order multiple pregnancies (HOM, more than three) are at greater risk for adverse maternal outcomes, perinatal morbidity and mortality, including multifoetal pregnancy loss, and may present the need for multifoetal pregnancy reduction (MFPR). The aim for multifoetal pregnancy reduction is to reduce the risk of maternal, foetal and perinatal complications, and it has been demonstrated to be justified, particularly in high-order multiple pregnancies (more than four foetuses). Multifoetal reduction increases the survival rates of the foetuses that remain [87].

The most frequent anomalies that are indicative for selective termination are central nervous system (CNS) anomalies for monochorionic twins and, likewise, CNS malformations and chromosomal anomalies in dichorionic twins [88]. Selective termination is a clinical procedure that can be performed in either the first or second trimester of gestation. The introduction of first-trimester screening reduces the time to diagnosis of chromosomal abnormalities and enhances the early diagnosis of many congenital abnormalities. The risk of foetal loss does not appear to increase if the chorionic villus sampling procedure is performed before the selective reduction [89,90,91]. The technique used for selective reduction changes according to the chorionicity of the pregnancy.

Foetal reduction in monochorionic pregnancies requires a technique that ablates or interrupts blood flow in the umbilical cord of the affected foetus. Due to the presence of vascular anastomoses in monochorionic pregnancies, it is impossible to use the injection of KCl because this would lead to the death of the co-twin; different techniques have been studied for this purpose. The currently used methods include bipolar cord coagulation (BCC) and radiofrequency ablation (RFA). The latter is a potentially less-invasive option, although recent papers do not report it to have an actual advantage in terms of preterm delivery or higher procedure-related loss [92,93].

In dichorionic twins, selective reduction can be performed transabdominally in the second trimester and transabdominally or transvaginally in the first. The technique involves the injection of potassium chloride (KCl) into the area of the foetal heart using a 21-gauge needle guided by an U.S. probe. The heart activity stops within 1 min after the injection. The activity must be monitored by ultrasound, and in rare cases, the injection can be repeated if necessary. Recent data suggest a significantly higher foetal loss if performed transvaginally rather than transabdominally [94].

A recent paper by Li *et al.* [95] reports an alternative method that involves an intracranial KCl injection in the first trimester. This technique appears to reduce the number of injections.

Selective foetal reduction in dichorionic twins is associated with excellent maternal and perinatal outcomes for the unselected co-twin. For monochorionic twins, the live birth rate is approximately 90% for BCC and ranges from 33%–90% for RFA, depending on the study [92,96]. Concerning the use of RFA, the studies are still few, with a limited number of reported cases.

Multifoetal pregnancy reduction is performed in the first trimester, and the technique is the same as that employed in selective termination in dichorionic pregnancies. The difference between the two is the decision of which foetus to terminate, and the choice depends on the position of the foetuses and their characteristics. The foetus with increased nuchal translucency (NT) is typically chosen. Enlarged NT is a risk factor for congenital abnormalities and genetic syndromes as well as chromosomal disorders; however, the choice should fall on the foetus with the larger NT, if it is possible to make this determination technically [97]. In fact, from a technical point of view, it is preferable to select the foetus nearest to the anterior uterine wall and furthest from the internal uterine orifice. Foetal loss is strongly related to the number of foetuses before and after the procedure, as it increases with the number of foetuses reduced.

## 6. Conclusions

The field of screening and prenatal diagnosis of twins is interesting yet poses many challenges. Within this particular field, the need of new, less invasive techniques is urgent. The highest priority, however, should be given to establishing, above all, the chorionicity of the pregnancy as well as the zygosity, with the utmost accuracy possible.
